# Total hip arthroplasty after rotational acetabular osteotomy for developmental dysplasia of the hip: a retrospective observational study

**DOI:** 10.1186/s12891-022-05597-y

**Published:** 2022-07-06

**Authors:** Takahiro Negayama, Ken Iwata, Masashi Shimamura, Teppei Senda, Tasuku Mashiba, Yoshio Kaji, Tetsuji Yamamoto

**Affiliations:** 1 Department of Orthopaedic Surgery, Faculty of Medicine, Kagawa University, 1750-1 Ikenobe, Miki-cho, Kita-gun, Kagawa, 761-0793 Japan; 2 Department of Orthopaedic Surgery, Saiseikai Kagawa Hospital, Kagawa, Japan

**Keywords:** Rotational acetabular osteotomy, Developmental dysplasia, Total hip arthroplasty, Bone defect, Hip center

## Abstract

**Background:**

Total hip arthroplasty after osteotomy is more technically challenging than primary total hip arthroplasty, especially concerning cup placement. This is attributed to bone morphological abnormalities caused by acetabular bone loss and osteophyte formation. This study aimed to investigate the clinical and radiological outcomes of total hip arthroplasty after rotational acetabular osteotomy compared with those of primary total hip arthroplasty, focusing mainly on acetabular deformity and cup position.

**Methods:**

The study included 22 hips that had undergone rotational acetabular osteotomy and 22 hips in an age- and sex-matched control group of patients who underwent total hip arthroplasties between 2005 and 2020. We analyzed cup abduction and anteversion; lateral, anterior, and posterior cup center–edge angle; hip joint center position; femoral anteversion angle; and presence of acetabular defect using postoperative radiography and computed tomography. Operative results and clinical evaluations were also analyzed.

**Results:**

The clinical evaluation showed that the postoperative flexion range of motion was lower in total hip arthroplasty after rotational acetabular osteotomy than in primary total hip arthroplasty, although no significant difference was noted in the postoperative total Japanese Orthopedic Association hip score. The operative time was significantly longer in the rotational acetabular osteotomy group than in the control group, but there was no significant difference in blood loss. The lateral cup center–edge angle was significantly higher and the posterior cup center–edge angle was significantly lower in the total hip arthroplasty after rotational acetabular osteotomy, suggesting a posterior bone defect existed in the acetabulum. In total hip arthroplasty after rotational acetabular osteotomy, the hip joint center was located significantly superior and lateral to the primary total hip arthroplasty.

**Conclusions:**

In total hip arthroplasty after rotational acetabular osteotomy, the cup tended to be placed in the superior and lateral positions, where there was more bone volume. The deformity of the acetabulum and the high hip center should be considered for treatment success because they may cause cup instability, limited range of motion, and impingement.

## Background

Developmental dysplasia of the hip (DDH) is the main cause of secondary hip osteoarthritis [1]. A variety of osteotomies are routinely performed due to the progression of osteoarthritis in younger patients when symptomatic DDH is left untreated [2]. Rotational acetabular osteotomy (RAO) is commonly used for the surgical treatment of symptomatic acetabular dysplasia in Japan [3]. Although many positive postoperative outcomes of RAO have been reported [4, 5], in some cases, the progression of osteoarthritis requires total hip arthroplasty (THA) as well [6, 7].

THA after osteotomy is more technically challenging than primary THA due to previous surgeries, abnormal bone morphology caused by bone defects, osteophyte formation, and other soft tissue problems, such as anatomical positional changes [8]. Several studies on THA after RAO have reported clinical results comparable to those of primary THA, although technical considerations are necessary [8, 9]. For example, bone grafting is often required due to bone loss, and subsequent postoperative cup migration has been reported [10], making THA after RAO more challenging than initial THA or THA after other osteotomies. Acetabular bone coverage is important for cup stability in THA after RAO owing to the tendency for bone defects in the anterior–posterior direction [11]. Previous studies have investigated acetabular defects in DDH by measuring the anteroposterior angle using computed tomography (CT) [12]; however, to the best of our knowledge, no study to date has investigated the acetabular defects in THA after osteotomy in the anteroposterior direction. This study aimed to determine the clinical outcomes and radiographic evaluation, including CT, of THA after RAO and compare them with those of primary THA.

## Methods

This was a retrospective observational study that compared two groups of patients. The ethics Committee of the University of Kagawa authorized the study design after all participants gave their informed permission (Approval code: 29–213). Between 2005 and 2020, THA was performed on 22 hips in 20 patients who had previously undergone RAO. RAO was performed in patients aged < 50 years with painful DDH up to Tönnis grade 1 [13]. One patient was excluded because he could not be reexamined for more than 1 year; thus, 21 hips of 19 patients were included in the study. THA in the left and right hips, in patients who underwent bilateral THA, was performed at different time points. For comparison, propensity score matching was used to identify 21 age- and sex-matched hips from 21 patients who had undergone THA for osteoarthritis secondary to DDH without undergoing prior hip surgery. These patients were included in the control group. Surgery was performed in 14 hips using the posterior approach and in seven hips using the direct anterior approach. Cementless stems and cups were used in all cases. In the RAO group, the following stems were used: S-ROM stems (DePuy Synthes, West Chester, PA, USA; 12 hips), Initia stems (Kyocera, Kyoto, Japan; four hips), Accolade II stems (Stryker Corp., Kalamazoo, MI, USA; two hips), Wagner cone stem (Zimmer Biomet, Warsaw, IN, USA; two hips), and Corail stems (DePuy Synthes; one hip); the following cups were used: Pinnacle cups (DePuy Synthes; 13 hips), SQRUM cups (Kyocera; four hips), Trident HA cups (Stryker; two hips), and Continuum cups (Zimmer Biomet; two hips). In the control group, J-taper stems (Kyocera; 18 hips) and S-ROM stems (DePuy Synthes; three hips), and SQRUM (Kyocera; 18 hips) and Pinnacle (Kyocera; three hips) cups were used. In patients with posterior wall defects of the acetabulum with the posterior cup center-edge (CE) (PCE) angle less than 0 degrees, the bulk bone was grafted; the extracted femoral head bone of the patient was used as the bone graft, prepared to fit the bone defect. In cases of bony impingement, the osteophytes of the acetabulum, anterior inferior iliac spine, and femoral greater trochanter were resected as much as possible. In patients who did not undergo bone grafting, hip range of motion training and full-load gait training began the day after surgery. In cases where bone grafting was used, partial loading began after 4 weeks of unloading. The operative time, blood loss, and complications obtained from medical records were reviewed.

The Japanese Orthopedic Association (JOA) hip score [14] was used to evaluate hip joint function preoperatively and at the final observation. The JOA hip score was assessed by 40 points for pain, 20 points for range of motion, 20 points for gait, and 20 points for activities of daily living, for a total of 100 points. Radiological evaluation included investigation of cup abduction and anteversion angle, cup CE angle, hip joint center position, femoral anteversion angle, and presence of acetabular defect. Cup inclination and anteversion angles were evaluated using radiography immediately after surgery.

**Fig. 1:**
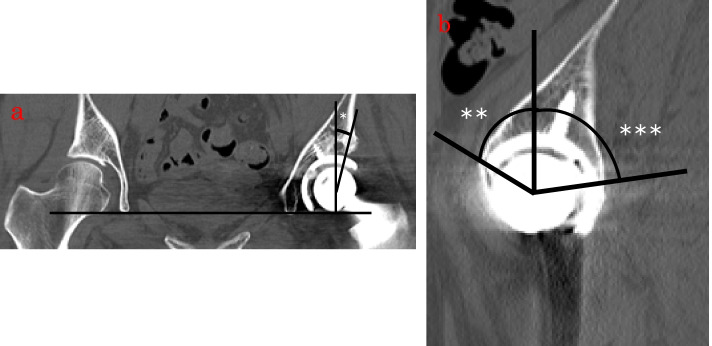
Postoperative computed tomography of THA. The LCE angle (*) is defined as shown in the postoperative coronal computed tomography image of THA (**a**). The ACE (**) and PCE angles (***) are defined as shown in the postoperative sagittal computed tomography image (**b**) THA: total hip arthroplasty; LCE: lateral cup center-edge; ACE: anterior cup center-edge; PCE: posterior cup center-edge

**Fig. 2:**
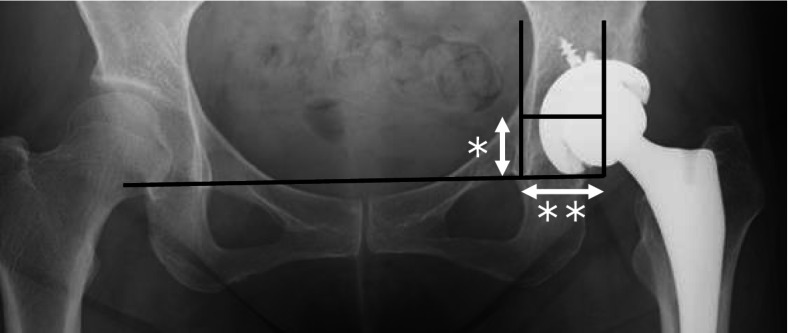
Postoperative anteroposterior X-ray radiogram of THA. The vertical distance (*) is defined as the distance from the lower edge of the bilateral the tear drops to the center of the hip joint. The horizontal distance (**) is defined as the distance of the horizontal direction from the lower edge of the tear drop to the hip joint center. THA: total hip arthroplasty

The lateral cup CE (LCE) angle was defined as the angle between the vertical line drawn from the center of the femoral head and the outer edge of the cup and acetabular contact using the coronal view of the CT of the hip (Fig. 1). The anterior cup CE (ACE) angle and the PCE angle were defined as the angles between the vertical line drawn from the center of the femoral head and the anterior and posterior edges of the cup and acetabular contact using a slice of the CT sagittal section at the center of the femoral head (Fig. 1). The hip joint center position was defined as the vertical and horizontal distance from the lower edge of the teardrop (Fig. 2) [15]. As the teardrop had moved after osteotomy, the position of the contralateral teardrop was used as a reference if no contralateral RAO was performed, and the CT finding before RAO was used as a reference if contralateral RAO was performed.

All analyses were performed using GraphPad Prism 9 software (GraphPad, San Diego, CA, USA). The mean and standard deviation of continuous variable distributions were reported. Frequencies and percentages were used to report categorical variables. The chi-square test was used to assess the statistical differences between the groups, while the unpaired t-test was used to examine the continuous outcomes. Statistical significance was set at *p* < 0.05. Differences between groups are reported with 95% confidence intervals (CIs).

## Results

### Patient demographics

Table 1 shows the demographics of the patients prior to surgery. There were no significant differences between the two groups in terms of age at THA, sex, follow-up duration after THA, or method. RAO and THA were separated by an average of 17.3 years.

**Table 1:** Patient demographics in the after RAO and control groups

	After RAO (*n* = 21)	Control (*n* = 21)	*P*-value
Number of patients (hips)	21	21	
Sex (male/female)	0/21	0/21	1.0
Interval between RAO to THA (years)	17.3 ± 7.2	N/A	
Follow-up period after THA (years)	7.1 ± 4.2	6.5 ± 1.9	0.54
Age at THA (years)	57.2 ± 9.2	60.6 ± 6.4	0.17
Surgical approach (PL/DA)	14/7	12/9	0.53

Data are presented as mean ± standard deviations or numbers

*RAO* Rotational acetabular osteotomy, *THA* Total hip arthroplasty, *PL* Posterior lateral, *DA* Direct anterior

### Operative results

The operative results are summarized in Table 2. The mean operative times were 173 and 130 min in the RAO and control groups, respectively. The RAO group had a significantly longer operative time than the control group (*p* < 0.001; 95% CI, 22.2–64.2 min)). The operative blood loss did not differ significantly between the two groups (*p* = 0.24).

**Table 2:** Operative results and clinical evaluation findings in the after RAO and control groups

	After RAO (*n* = 21)	Control (*n* = 21)	*P*-value
Operative time (min)	172.1 ± 34.0	130.1 ± 23.2	< 0.001
Operative blood loss (g)	346.8 ± 215.8	312.1 ± 247.9	0.24
JOA score (preoperative)			
Total (points)	40.7 ± 9.2	40.2 ± 6.4	0.37
Pain (points)	6.7 ± 5.6	7.2 ± 4.5	0.88
ROM (points)	12.2 ± 3.1	11.5 ± 3.9	0.35
Gait (points)	10.3 ± 3.0	10.8 ± 2.8	0.88
Activity of daily living (points)	11.6 ± 2.1	10.7 ± 1.2	0.06
JOA score (last follow-up)			
Total (points)	93.0 ± 3.7	95.6 ± 4.2	0.13
Pain (points)	38.5 ± 2.3	39.2 ± 1.9	0.67
ROM (points)	16.4 ± 2.1	17.9 ± 1.6	0.03
Gait (points)	19.0 ± 1.4	19.3 ± 2.3	0.46
Activity of daily living (points)	19.1 ± 1.2	19.2 ± 1.4	0.90
ROM (preoperative)			
Flexion (°)	80.0 ± 13.1	86.6 ± 6.9	0.08
Extension (°)	-2.2 ± 3.0	-3.6 ± 5.1	0.30
Abduction (°)	19.7 ± 7.0	17.9 ± 9.1	0.50
Adduction (°)	12.2 ± 3.0	9.5 ± 5.8	0.09
ROM (last follow-up)			
Flexion (°)	92.2 ± 6.8	99.2 ± 8.3	0.02
Extension (°)	-0.5 ± 1.2	-0.3 ± 1.1	0.16
Abduction (°)	28.6 ± 3.2	30.3 ± 3.4	0.15
Adduction (°)	9.2 ± 2.4	10 ± 3.2	0.40
Bone grafting	2 (9.5)	0 (0)	0.15

Data are presented as mean ± standard deviations or numbers (%)

*RAO* Rotational acetabular osteotomy, *JOA Japanese Orthopedic Association, ROM* Range of motion

**Fig. 3:**
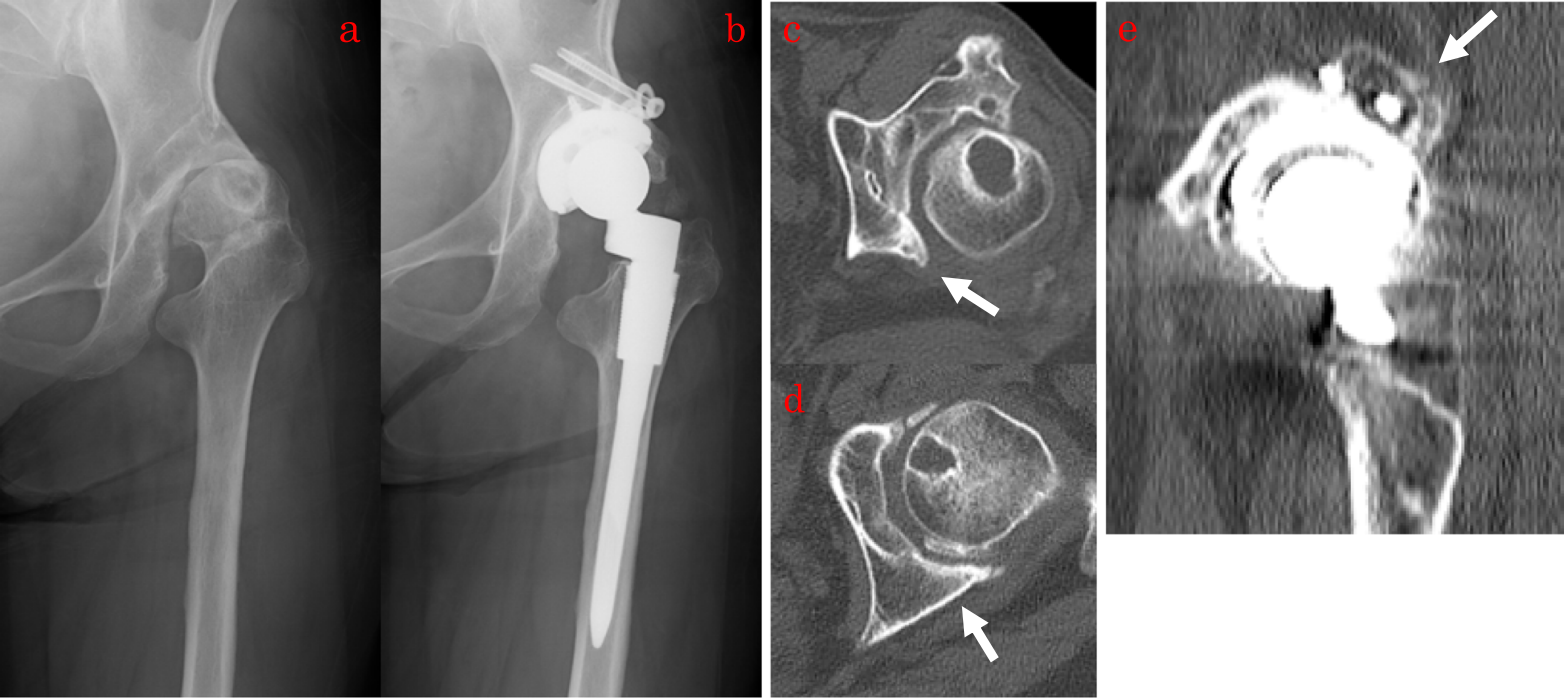
Preoperative and postoperative X-ray and CT images of THA after RAO. Preoperative and postoperative anteroposterior radiographs of THA for a patient with hip osteoarthritis who underwent RAO 20 years ago (**a**,** b**). This is the axial section of CT image. Defects in the posterior wall of the acetabulum are observed (arrow) (**c**). This is the axial section of a CT image of a patient in the control group. There is no bone defect of acetabulum (arrow) (**d**). This is the sagittal section of a CT image. Bulk bone graft (arrow) was performed because of the fear of insufficient fixation of the cup due to bone defect in the posterior wall of the acetabulum (**e**). CT: computed tomography; RAO: rotational acetabular osteotomy; THA: total hip arthroplasty

The RAO group’s JOA hip score increased from 40.7 points preoperatively to 93.0 points postoperatively, while the control group’s JOA hip score increased from 40.2 points preoperatively to 95.6 points postoperatively. Between the two groups, there was no significant difference in total postoperative JOA hip scores (*p* = 0.13). The ROM values at the final follow-up examination were 16.4 and 17.9 points in the RAO and control groups, respectively. There was a significant difference in the ROM at the final follow-up examination between the two groups (*p* < 0.03; 95% CI, -2.87– -0.15 points). The flexion range of motion at the final follow-up examination was lower in the RAO group than that in the control group (*p* < 0.02; 95% CI, -12.8–1.2°), although no significant differences were noted in extension, abduction, or adduction. Two patients in the RAO group required bulk bone grafting at the posterior wall of the acetabulum (Fig. 3).

### Radiographic findings

The radiographic findings are summarized in Table 3. There was no significant difference in cup inclination (*p* = 0.58) and anteversion angle (*p* = 0.24) between the two groups. The LCE angle was significantly different between the two groups (*p* = 0.01; 95% CI, 1.5–10.8°), with a 35.5° angle in the RAO group and a 29.3° angle in the control group. The PCE angle was also significantly different between the two groups (*p* < 0.001; 95% CI, -80– -45.5°), with a 44.4° angle in the RAO group and a 107.2° angle in the control group. There was no significant difference in the ACE angle between the two groups *(p* = 0.15). These data suggest that the RAO group exhibited more bony coverage laterally but less bony coverage posteriorly than the control group. The position of the center of the hip joint differed significantly in vertical distance (*p* < 0.001; 95% CI, 4.05–12.6 mm); the vertical distance was 29.6 mm in the RAO group and 22.3 mm in the control group. There was also a significant difference in the horizontal distance between the two groups (*p* = 0.002; 95% CI, 2.45–9.65 mm), being 35.8 mm in the RAO group and 29.7 mm in the control group. The cup placement was higher and lateral in the RAO group compared with that in the control group. The femoral anteversion angle did not differ significantly between the two groups preoperatively *(p* = 0.30) or postoperatively *(p* = 0.20). In both the groups, there were no complications of fracture, dislocation, infection, or neurovascular injury.

**Table 3:** Radiographic findings in the after RAO and control groups

	After RAO (*n* = 21)	Control (*n* = 21)	*P*-value
Cup angle			
Inclination (°)	36.3 ± 5.3	36.8 ± 3.5	0.58
Anteversion (°)	17.9 ± 9.6	21.4 ± 5.2	0.24
Cup CE angle			
LCE (°)	35.5 ± 4.8	29.3 ± 7.9	0.01
ACE (°)	64.4 ± 6.0	59.9 ± 8.4	0.15
PCE (°)	44.4 ± 26.7	107.2 ± 12.0	< 0.001
Hip joint center			
Vertical distance (mm)	29.6 ± 7.2	22.3 ± 4.8	< 0.001
Horizontal distance (mm)	35.8 ± 6.0	29.7 ± 4.2	0.002
Femoral anteversion			
Preoperative (°)	33.6 ± 17.5	29.2 ± 10.1	0.30
Postoperative (°)	41.2 ± 12.9	34.5 ± 10.3	0.20

Data are presented as mean ± standard deviations

*RAO* Rotational acetabular osteotomy, *LCE* Lateral cup CE, *PCE* Posterior cup CE, *ACE* Anterior cup CE

## Discussion

This study revealed that the postoperative JOA score of THA after RAO was comparable to that of the control group. In addition, radiologically, the RAO group showed increased lateral coverage of the acetabulum and characteristic bone defect in the posterior wall of the acetabulum because of the excessive anterior rotation of the osteotomy fragment. Hence, the cup was required to be positioned on the upper and lateral sides where there was more bone mass, which may have caused limited range of motion and bony impingement.

RAO is a joint-preserving surgery in which the acetabulum is osteotomized into a spherical shape and rotated laterally to increase the coverage of the femoral head by the acetabulum to improve joint congruity. It is commonly performed in young and adolescent patients with DDH [16, 17]. While positive postoperative outcomes of RAO have been reported [4], there are cases of advanced osteoarthritis leading to THA [18, 19]. Whether a previous RAO has an impact on the clinical outcomes of subsequent THA remains controversial. Interestingly, previous studies have reported no significant differences in postoperative clinical, operative time, blood loss, or cup inclination angle [20, 21]. In contrast, studies have reported that THA after RAO decreases the postoperative Harris hip score and patient satisfaction compared with primary THA [22, 23]. Furthermore, it has been reported that THA after RAO is more challenging to perform due to bone deformity and the operative time is significantly longer than that of primary THA [8, 24]. As mentioned above, whether a previous RAO can affect clinical outcomes remains unknown, but cup placement is difficult and may affect the operative time and radiographic evaluation. In addition, bone defects in the posterior acetabular wall with large osteophytes are factors that complicate THA after RAO when compared with that after Chiari osteotomy and shelf acetabuloplasty [10]. As shown in these reports, bone defects in the acetabular wall are characteristics of RAO. However, there are no reports that have examined bone coverage in the anterior–posterior direction in CT after THA, as the ACE and PCE angles. In this study, the osteotomy fragments were rotated anteriorly to increase the anterior bony coverage, resulting in a significantly lower PCE angle than that in the control group; thus, the posterior acetabular bone defect was more likely to occur. It has been reported that in THA after RAO, the cup was often placed more laterally compared to that in primary THA [8]. When comparing the RAO to the control group, the hip center was laterally and superiorly positioned in the RAO group. This was thought to be the result of placing the cup in the superolateral region, where there was more bone volume, according to the shape of the RAO acetabulum. These results suggest that the posterior acetabular bone defect and cup position should be carefully considered when performing THA after RAO. Regarding the angle of cup placement, osteosclerosis and osteophytes around the acetabulum after osteotomy can make the surgery more difficult [9], and acetabular retroversion and posterior wall defects can reduce the cup anteversion angle [25, 26]. We have addressed these limitations by performing careful preoperative surgical planning and adequate fluoroscopic confirmation of the cup angle. In addition, the direct anterior approach was used in some cases, which allowed more accurate placement of the cup by stabilizing the pelvis in the supine position and using a cup alignment guide based on the anterior superior iliac spine.

Bulk bone grafting is useful for acetabular defects [27, 28]; however, dislocation of the grafted bone can occur in bulk bone grafting for bone defects after RAO [10]. We performed bulk bone grafting for a posterior acetabular bone defect, and the grafted bone survived without any postoperative dislocation. While it is necessary for the cup to be placed in an area of high bone mass to obtain good initial fixation, care should be taken in high placement. Elevating the center of the hip increases the bone coverage of the cup, but it also decreases the range of motion and is a risk factor for dislocation and THA failure [29–31]. Although the cup needs to be placed as close to the original acetabulum as possible, there is no significant difference in clinical outcomes or implant survival if the center of the head is not higher than 35 mm above the inferior edge of the teardrop in primary THA [32]. We allowed slight elevation of the center of the hip, as we thought that it was better to fix the cup without bone grafting in the superior part of the loading area to achieve cup stability. However, when performing THA after RAO, the osteotomy fragment is rotated more anteriorly. Thus, the possibility of impingement of the anterior acetabular osteophyte, cup, or anterior inferior iliac spine with the femur is even higher with a high hip center. Therefore, careful preoperative planning and intraoperative confirmation of impingement and resection of the impinging bone are necessary. This bony impingement could be the reason for the poorer postoperative range of motion in the THA after RAO group compared with that in the control group. Although it is necessary to reduce the leg length difference as much as possible when elevating the hip center, the two patients with bilateral THA in this study had good postoperative outcomes with no leg length difference.

Although it has been known that bone defects in the anterior and posterior acetabular walls occur after RAO, to the best of our knowledge, this study is the first to evaluate bone defects in the anterior and posterior acetabular walls when performing THA after RAO using sagittal sections of CT. Since the PCE angle is significantly reduced in THA after RAO, the posterior defect of the acetabulum and cup stability should be carefully considered when performing THA.

However, there were various limitations in this study. First, this was a retrospective study and patients were not randomized. There was no significant difference in patient background in terms of sex, age, follow-up period after THA, or surgical approach, although there could be bias because of unmeasured factors. Second, only a few years had passed since surgery in some cases of patients and, therefore, long-term follow-up data of such patients are not yet available. Because of the strong deformation of the acetabulum in these patients compared to those with primary THA, we will continue to follow-up the survival rate of THA, especially the cup survival rate, dislocation rate, and clinical evaluation over time.

## Conclusion

The clinical results of THA after RAO were comparable to those of primary THA. Preoperative planning should be tailored to the acetabular deformity with attention to bone defects in the posterior wall of the acetabulum and dislocation due to impingement.

## Data Availability

The datasets used and/or analyzed during the current study available from the corresponding author on reasonable request.

## Abbreviations

DDH: Developmental dysplasia of the hip RAO
THA: Total hip arthroplasty
CT: Computed tomography
JOA: Japanese Orthopaedic Association
CE: Center-edge
LCE: Lateral cup center-edge
ACE: Anterior cup center-edge
PCE: Posterior cup center-edge
